# Conservation and divergence within the clathrin interactome of *Trypanosoma cruzi*

**DOI:** 10.1038/srep31212

**Published:** 2016-08-09

**Authors:** Ligia Cristina Kalb, Yohana Camila A. Frederico, Cordula Boehm, Claudia Maria do Nascimento Moreira, Maurilio José Soares, Mark C. Field

**Affiliations:** 1 Laboratory of Cell Biology, Instituto Carlos Chagas/Fiocruz-PR, Rua Prof. Algacyr Munhoz Mader 3775, Cidade Industrial, 81350-010 Curitiba, PR Brazil; 2 Laboratory of Molecular Biology of Trypanosomes, Instituto Carlos Chagas/Fiocruz-PR, Rua Prof. Algacyr Munhoz Mader 3775, Cidade Industrial, 81350-010 Curitiba, PR Brazil.; 3 School of Life Sciences, University of Dundee, Dundee, DD1 5EH, UK

## Abstract

Trypanosomatids are parasitic protozoa with a significant burden on human health. African and American trypanosomes are causative agents of Nagana and Chagas disease respectively, and speciated about 300 million years ago. These parasites have highly distinct life cycles, pathologies, transmission strategies and surface proteomes, being dominated by the variant surface glycoprotein (African) or mucins (American) respectively. In African trypanosomes clathrin-mediated trafficking is responsible for endocytosis and post-Golgi transport, with several mechanistic aspects distinct from higher organisms. Using clathrin light chain (TcCLC) and EpsinR (TcEpsinR) as affinity handles, we identified candidate clathrin-associated proteins (CAPs) in *Trypanosoma cruzi;* the cohort includes orthologs of many proteins known to mediate vesicle trafficking, but significantly not the AP-2 adaptor complex. Several trypanosome-specific proteins common with African trypanosomes, were also identified. Fluorescence microscopy revealed localisations for TcEpsinR, TcCLC and TcCHC at the posterior region of trypomastigote cells, coincident with the flagellar pocket and Golgi apparatus. These data provide the first systematic analysis of clathrin-mediated trafficking in *T. cruzi,* allowing comparison between protein cohorts and other trypanosomes and also suggest that clathrin trafficking in at least some life stages of *T. cruzi* may be AP-2-independent.

Transfer of proteins and lipids between intracellular compartments by vesicular transport is a fundamental process and central to many eukaryotic cellular functions^1^. Multiple compartments and pathways comprise the exo- and endocytic arms of the endomembrane system. Transport between these compartments involves budding of protein-coated vesicles from donor membranes, a process essential for cargo sorting^2^. One of the best characterised coat proteins is clathrin^3^,^4^. Assembly of clathrin into lattices in higher eukaryotes serves to select cargo proteins, in part by incorporation of cargo receptor complexes and proteins into the growing clathrin coat. Lattice formation also facilitates membrane deformation and clathrin participates in sorting at the plasma membrane, endosomes and *trans* face of the Golgi complex, contributing in a wide range of individual sorting and transport events^5^,^6^.

In Saccharomyces *cerevisiae* over 60 proteins are transiently associated with endocytic sites, in a highly dynamic and orchestrated process consistent with clathrin-mediated endocytosis (CME) as tightly regulated and modular^7^,^8^. Similarly, in mammalian cells over 40 proteins are recruited in a precise sequence to CME sites^9^. A network initially assembles around FCHO proteins, phosphatidylinositol 4,5-phosphate and receptors at the plasma membrane, and rapidly recruits adaptor proteins including DAB2, eps15 and intersectin^10^. AP complexes, Epsin, AP180 and many other cargo receptors are incorporated into the clathrin lattice. Dynamins are recruited by the accessory proteins amphiphysin, sorting nexin-9 and/or intersectin to the neck of the vesicle to enact membrane scission on GTP hydrolysis, whereas auxilin and the ATPase Hsc70 are involved in clathrin uncoating. The CME protein requirement is variable between cell types, suggesting adaptation to the ligands endocytosed and specific dynamic requirements, although the precise relationships between the proteins mediating CME and function are not always clear^8^.

In trypanosomatids, a group of pathogenic protozoa afflicting much of the world’s population, clathrin-based trafficking represents an important interface with the host and plays multiple roles in immune evasion and host cell invasion vital for effective infection and persistence^11^. The American trypanosome, *Trypanosoma cruzi,* is both a hemoflagellate and intracellular pathogen and causes Chagas disease in South and Central America^12^. All evidence is consistent with clathrin-mediated endocytosis (CME) being restricted to the flagellar pocket, a common feature of trypanosomatids^13^.

Membrane transport is well characterised in African trypanosomatids and lacks multiple proteins that are otherwise widely conserved. This includes the AP-2 complex, a major mediator of clathrin sorting in endocytic systems many organisms^14^,^15^,^16^. More broadly, several proteins, including FCHO, Epsin and several monomeric adaptor proteins are restricted to animals or animals and fungi. These divergent features result in a predicted clathrin network for trypanosomes that is rather sparse, suggesting either massive simplification, extreme sequence divergence preventing *in silico* identification or the presence of alternate components^16^. Significantly, many clathrin-associated proteins, or CAPs, are present in parasitic protozoa, of which several are trypanosomatid specific^17^,^18^,^19^.

Many observations indicate the presence of distinct compartments and structures within the *T. cruzi* endomembrane system which are distinct from African relatives, indicating that comparative analysis between trypanosomes is of significance. For example a feature differentiating *T. brucei* and *T. cruzi* is clathrin-independent endocytosis, that in the latter operates mainly through the cytostome/cytopharynx^20^,^21^. This structure is an invagination of the plasma membrane close to the flagellar pocket and which penetrates deep into the cytoplasm, frequently terminating at the posterior end of the cell and distal to the nucleus^22^,^23^,^24^. Interestingly, clathrin is found at the contractile vacuole complex in *T. cruzi*^25^ similar to *Dictyostelium discoideum*^26^,^27^, while AP180, a clathrin assembly protein, is also present in *T. cruzi* clathrin coated vesicles^25^. Uptake of extracellular material is restricted to the flagellar pocket and the cytostome in epimastigotes^28^,^29^, but in trypomastigotes, which lack a cytostome, endocytosis appears to be largely absent^30^. Molecules ingested through the cytostome are internalized by endocytic vesicles, and it has been proposed that cargo enters the cytostome and passes through an early endosomal network before storage or degradation in reservosomes^29^. However, it has been also suggested that endocytic vesicles derived from the cell surface transfer their contents directly to the reservosome without passing through any intermediate compartments^31^. The presence of orthologs of Rab proteins associated with early and intermediate endosomes of other organisms in *T. cruzi* argues for a complex endomembrane system, and this matter has yet to be resolved.

Overall, these observations indicate considerable morphological and mechanistic divergence between the trafficking systems of trypanosomes and their hosts within trypanosome lineages. Here we characterised the clathrin interactome of *T. cruzi* using affinity isolation/proteomics in epimastigotes expressing fusion protein forms of clathrin light chain or EpsinR. Over 30 distinct proteins were identified, several of which are novel and/or trypanosome-specific. These data provide the first proteomic analysis of clathrin-mediated trafficking in *T. cruzi* and allow a detailed comparison of this protein cohort with other trypanosomes and the host.

## Results

### Isolation of clathrin-interacting proteins from *Trypanosoma cruzi*

To initiate a systematic and unbiased identification of proteins interacting with the clathrin in *T. cruzi* we created transgenic epimastigotes harbouring epitope-tagged forms of the clathrin light chain (CLC) and EpsinR, both of which interact with the clathrin heavy chain. Both were tagged at the N-terminus, and expressed in cells as GFP::TcEpsinR or Protein A::TcCLC.

**Figure 1:**
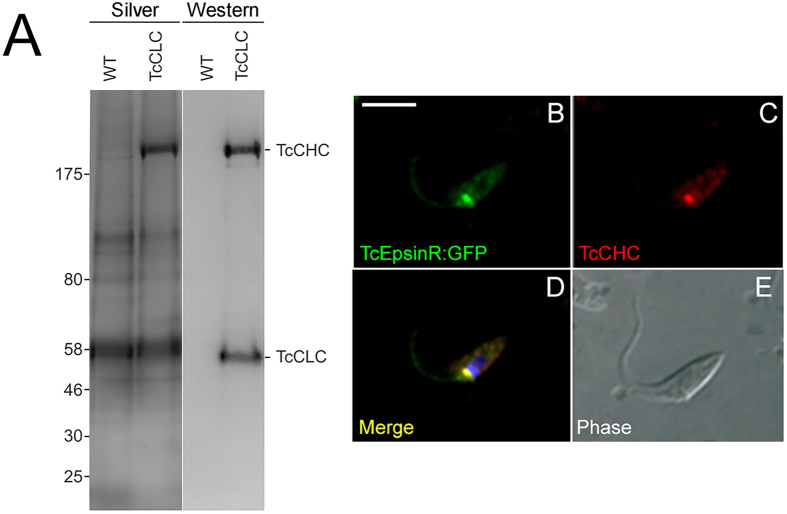
Immunoprecipitation of *T. cruzi* clathrin-associated proteins (TcCAPs). Panel (**A**) Protein complexes isolated by immunoprecipitation from cryolysates of *T. cruzi* epimastigotes expressing Protein A:TcCLC (+) using Dynabeads M280 coupled to sheep anti rabbit-IgG were resolved by 4–12% gradient SDS-PAG. Wild-type cell lysate (WT) was used as a negative control. Coomassie staining showed the presence of a prominent 192 kDa band (TcCHC), but not in the negative control. Visualization of TcCHC (192 kDa) was by reaction with a monoclonal antibody against TcCHC and the visualization of TcCLC/AC (55k Da) was by reaction with an anti-rabbit secondary antibody, which has affinity for protein A. Panels (**B–E**) Immunocolocalization of clathrin heavy chain (TcCHC) and TcEpsinR in *Trypanosoma cruzi* epimastigotes. Nucleus and kinetoplast DNA were stained with Hoechst 33342.Transfected epimastigote expressing EpsinR-GFP incubated with antibody against GFP (TcEpsinR) and TcCHC monoclonal antibody (clathrin). Note co-localization of the GFP and TcCHC signals (**D**). (**E**) Differential interference contrast (DIC) image of the parasite body. Scale bar 5 μm. Images are representative of n = 10 cells.

Initially, using TcCLC as affinity handle, coupled with cryomilling, we identified a large cohort of candidate interacting proteins using label-free proteomics. Cryomilling provides a robust method by which one can preserve protein-protein interactions in the cell and has been applied to many organisms and systems (see Obado *et al.*, 2016 for an example in trypanosomes). Analysis of these complexes by 1D SDS-PAGE and visualisation by Silver staining indicated multiple co-isolated proteins (Fig. 1). Significantly, a prominent band was observed at ~200 kDa in the electrophoretogram, and which was subsequently identified as the clathrin heavy chain by Western blotting with monoclonal antibody to TcCHC^21^ and subsequently by mass spectroscopy (Fig. 1A, Table 1). Neither TcCLC or TcCHC were detected in control isolates. Following mass spectrometric analysis of these isolations and comparisons with the untagged control, we observed that the affinity-tagged isolations included both conserved and novel clathrin-associated proteins (CAPs) (Table 1). Similar protein profiles were obtained in two independent immunoprecipitations for TcCLC and three for TcEpsin, indicating that the isolation procedure was reproducible and thus likely robust.

Peptide sequences predicted by MS were used to query the *T. cruzi* predicted proteome in order to identify proteins that copurified with Protein A::TcCLC. Besides TcCHC (TcCLB.506167.50), over 30 additional proteins were identified (Table 1). Amongst these were TcEpsinR, subunits of the AP-1 and AP-4 complexes and AP180. We applied a cutoff criterion of five-fold greater emPAI score in the test versus the control isolation, together with an exclusion of 0.1 emPAI (see Supplementary data for full MS reporting). The vast majority of proteins was identified in both replicates, with the exception of some low abundance SNARE and Rab proteins and dynamin (TcCLB.508153.20). This latter protein is a frequent contaminant in membrane fractions^32^ and whilst it may be involved in endocytic functions, it is unclear from these data.

The second highest ranked protein in the TcCLC isolation was the *T. cruzi* ortholog of EpsinR. Tagging of this protein with GFP at the N-terminus to produce GFP::TbEpsinR and immunofluorescence using anti-GFP and anti-clathrin heavy chain monoclonal antibody demonstrated significant colocalisation for these two proteins, at the anterior region of the cell and close to the flagellar pocket (Fig. 1B). Whilst the resolution of light microscopy is insufficient to confirm a direct interaction, these data do indicate that TcEpsinR and TcCLC have the potential to interact, based on proximity, and provides additional support for this connection. This is also consistent with previous work in *T. brucei*^18^.

### Isolation of TcEpsinR-interacting proteins from *Trypanosoma cruzi*

**Figure 2:**
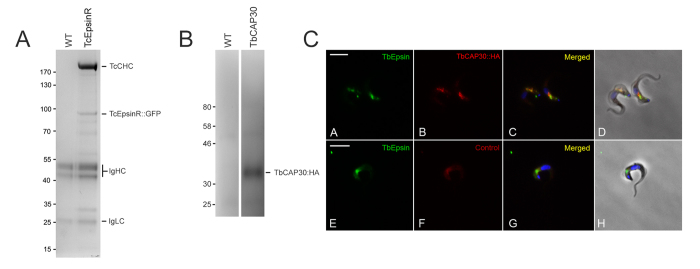
Immunoprecipitation of TcEpsinR-interacting proteins. (Panel **A**) Protein complexes isolated by immunoprecipitation from *T. cruzi* epimastigotes expressing GFP::TcEpsinR (lane2) using Dynabeads M270 coupled to llama anti-GFP and resolved 4–12% SDS-PA. Wild-type cell lysate (WT) was used as control. Coomassie staining showed the presence of the 192 kDa TcCHC, but not in the control. (Panel **B**) Correct tagging of TbCAP30. WT: wild forms of *T. brucei*. TbCAP30: protein extract of *T. brucei* bloodstream forms expressing TbCAP30::HA (Gene ID Tb927.8.7230, 30 kDa). Analysis with anti-HA antibody showed reaction with a polypeptide with molecular mass (33 kDa) compatible with that predicted from the gene sequence in *T. brucei* (30 kDa) plus an HA-tag (3 kDa). (Panel **C**) Immunocolocalization of Tb927.8.7230 and TbEpsinR in *Trypanosoma brucei* bloodstream forms. Transfected bloodstream forms expressing Tb927.8.7230 fused with HA incubated with rat anti-HA antibody (**B,F**) and TbEpsinR polyclonal rabbit antibody (**A,E**); note partial co-localization of the HA and TbEpsinR signals (**C,G**). Nucleus and kinetoplast DNA were stained with Hoechst 33342 (**C,G**). Differential interference contrast (DIC) images of the parasite body (**D,H**). Scale bar 5 μm.

**Figure 3:**
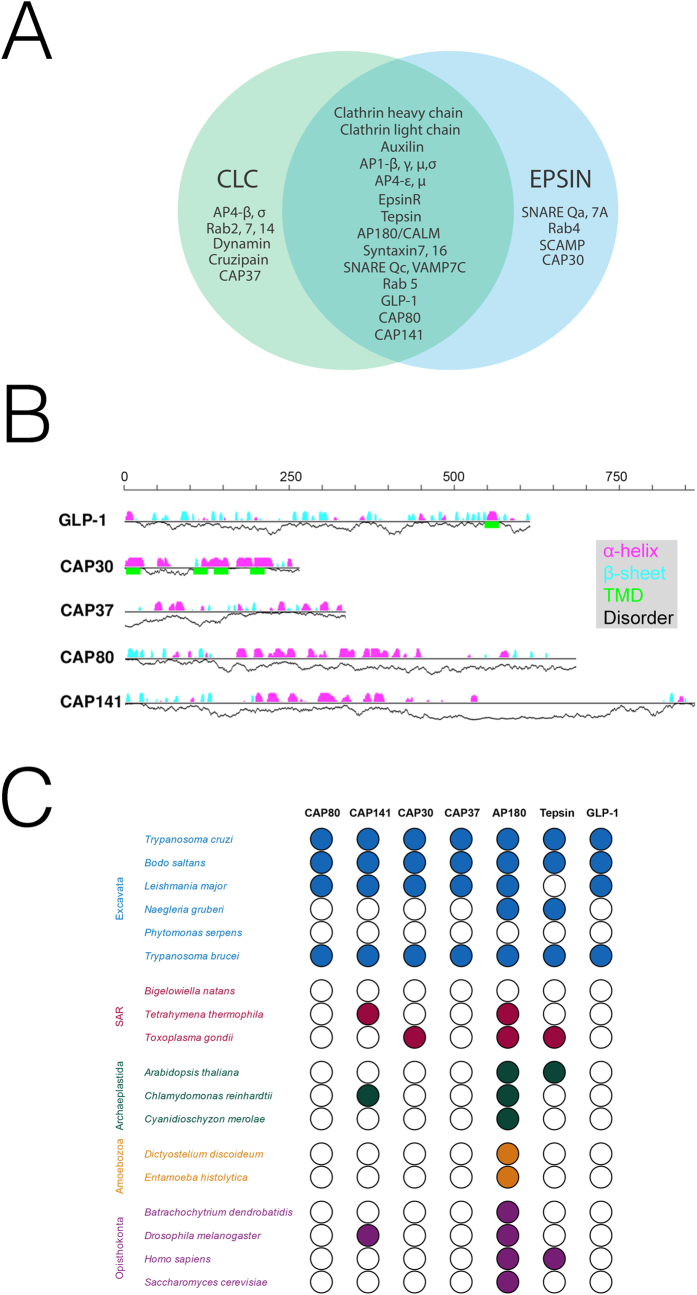
Proteins identified by TcCLC and TcEpsinR. (Panel **A**) Venn diagram of the most significant proteins identified with either GFP::TcCLC or Protein A:TcEpsinR. See also Tables 1 and 2 for statistical data and supplementary data for full information. (Panel **B**) Predicted secondary structures of GLP-1 and TcCAP30, 30, 80 and 141. α and β secondary structure probability is indicated above the line in purple or cyan respectively. *Trans*-membrane domains and disorder probability are shown below the lines in green and as a black line respectively. The scale bar is protein length in amino acid residues. (Panel **C**) Coulson plot of novel proteins identified by proteomics. The genomes of select taxa were searched using reciprocal BLAST, together with manual inspection of the alignment as a test for the presence of an ortholog. Filled circles indicate that a high confidence ortholog was found, and open circles indicate that an ortholog was not identified.

To strengthen the evidence that the proteins identified by immuno-isolation of TcCLC complexes are genuine clathrin interaction partners, a reciprocal co-immunoprecipitation was performed using GFP::TcEpsinR. Immunoprecipitation of GFP::TcEpsinR using magnetic beads covalently coupled to llama anti-GFP antibody successfully co-precipitated clathrin heavy and light chains from tagged *T. cruzi* epimastigotes (Fig. 2A). Again LCMS^2^ was used to identify the proteins in these complexes using three replicates, and besides TcCHC (TcCLB.506167.50), over 30 additional proteins were confidently identified (Table 2, Fig. 3).

### A cohort of endocytic proteins in *T. cruzi*

It is significant that a great many proteins identified using GFP::CLC and Protein A::EpsinR were in common (Fig. 3). This orthogonal identification supports the hypothesis that these are indeed *bona fide* endocytic proteins in *T. cruzi*. Of these, TcCHC was recovered from all five isolations (two × GFP::CLC and three × Protein A::EpsinR) while TcCLC was also found in all three TbEpsinR isolates. The ortholog of AP180/CALM (TcCLB.503449.30) was recovered from four of five experiments. Together with TcEpsinR these proteins are involved in AP-2-independent clathrin-mediated endocytosis in *T. brucei*^13^, and the data here suggest a similar configuration in *T. cruzi*. A clathrin-uncoating protein, the trypanosome auxilin ortholog (TcCLB.510045.30) was also found in four of five independent experiments.

Four candidate clathrin-associated proteins (CAPs) encoded by TcCLB.503595.10 (TcCAP80), TcCLB.507221.70 (TcCAP141), TcCLB.510057.30 (TcCAP37) and TcCLB.507895.170 (TbCAP30) all encode hypothetical proteins (Fig. 3). Apart from a similar structure of predominantly β-sheet at the N-terminus and disordered/α-helical at the C-terminus for TcCAP80 and TcCAP141, these proteins appear quite divergent in secondary structure. All are essentially restricted to trypanosomatids, and even absent from the heterolobosid *Naegleria gruberi,* a sister lineage (Fig. 3). Orthologs of TcCAP80 and TcCAP141 have also been identified in *T. brucei* through affinity isolat using the TbCHC as the affinity handle, and mediate endocytosis and morphological features of the flagellar pocket (Manna *et al.*, 2016 submitted), suggesting that this cohort are also likely *bona fide* players in endocytosis in *T. cruzi*.

Two heterotetrameric adaptor complexes were recovered with both affinity handles, the AP-1 (TcCLB.508257.260, TcCLB.510533.40, TcCLB.506247.200 and TcCLB.509623.19), which is involved in clathrin-mediated traffic from the Golgi complex and the AP-4 (TcCLB.511751.200, TcCLB.509911.70, TcCLB.504137.60 and TcCLB.506525.104). Significantly, we also recovered Tepsin (TcCLB.504105.120), a central component of AP-4-containing vesicles^33^. This protein is broadly conserved and present in most kinetoplastids except for the *Phytomonas* and *Leishmania* lineages, which significantly also lack the AP-4 complex, evidence that Tepsin is likely also associated with AP-4 in trypanosomatids^34^. In addition, Tepsin represents an additional member of the ANTH/ENTH family of phosphoinositide-binding trafficking proteins, beyond those characterised so far in trypanosomes, i.e. TbEpsinR and TbCALM.

**Figure 4:**
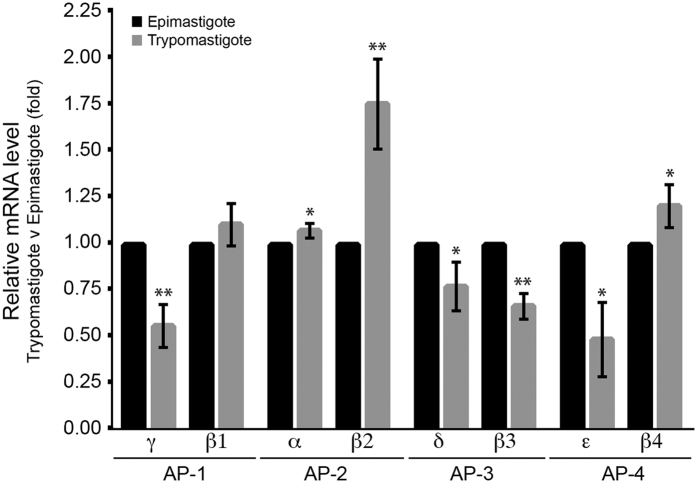
Relative mRNA expression of heavy chain subunits of adaptor complexes AP-1 to 4 in trypomastigote and epimastigote forms of *T. cruzi.* Data normalization for RNA was relative to the telomerase reverse transcriptase (TERT) gene. Epimastigote form level was set at 1.0 and data are presented as mean (±SD). Data analyses were performed as Livak and Schmittgen, 2001. The asterisks represent significant (*p < 0.05) or very significant (**p < 0.01) expression level differences between two life stages of each adaptin gene of *T. cruzi*. The experiment was performed in technical triplicate. The sequences of oligonucleotides used in this analysis are given in Table S1.

Unexpectedly, we found no evidence in any of our isolations for AP-2, the adaptin complex that in higher eukaryotes associates with clathrin at the plasma membrane. In African trypanosomes this entire complex is absent from the genome^34^, but all subunits are present in the *T. cruzi* genome. A trivial explanation is that AP-2 is simply down-regulated in the epimastigote stage. To at least partially approach this question, we analysed the mRNA levels of AP-2 transcripts in epimastigotes and trypomastigotes using qRT-PCR (Fig. 4). AP-2 mRNA was easily detected in both of these life stages, and which is also consistent with a recent transcriptome study of *T. cruzi*^35^. Therefore, it appears that the failure to capture AP-2 in these pullouts is unlikely due simply to an absence of expression, and raises the possibility that CME in *T. cruzi* epimastigotes is, similarly to *T. brucei*, also AP-2 independent.

Five Rab proteins were recovered. TcCLB.509805.60 (Rab5) was recovered by both TcCLC and TcEpsinR; TcCLB.511621.120 (Rab14), TcCLB.508461.270 (Rab7) and TcCLB.511711.80 (Rab2) were isolated only for TcCLC and Rab4 (TcCLB.510911.30) only for TcEpsinR. We also recovered seven SNAREs: TcCLB.506855.140 (SNARE Vamp7c), TcCLB.507795.50 (Syntaxin 7), TcCLB.508465.120 (Syntaxin 16), TcCLB.508955.10 (Qc SNARE) from both TcCLC and TcEpsinR and TcCLB.511627.60 (SNARE VAMP7a), TcCLB.507811.60 (SNARE Vamp7b), TcCLB.506401.130 (Qa-SNARE) only in the TcEpsinR list. Several proteins that are likely cargo, i.e. TcCLB.511391.180, which encodes GLP-1, and TcCLB.507537.20 that encodes cruzipain, were also recovered using both affinity handles (Fig. 3). Finally we also recovered the product of TcCLB.509319.40, a *trans*-membrane-domain protein that is associated with the Golgi complex in *S. cerevisiae.* Significantly orthologs of TcCLB.509319.40 are widely distributed across eukaryotes.

### *Localisation* of *TcCAP30*

From the TcEpsinR isolation we selected the hypothetical protein TcCLB.507895.170, on account of its apparent novelty as a candidate clathrin-associated protein in this protozoan, the fact that it has not previously been localied (unlike CAP80 and CAP141, where this has been done in *T. brucei* (Manna *et al.*, 2016 under revision)) and exclusive presence in trypanosomatids. However, it was more convenient to investigate this protein in *T. brucei* (Tb927.8.7230: TbCAP30, 30 kDa) bloodstream forms, where clathrin localizes to endomembrane compartments restricted to the region between the kinetoplast and nucleus. As the general organisation of the endosomal system of *T. cruzi* is similar, we anticipated that *bona fide* CAP proteins should localize to this region. We determined the location of the gene product TbCAP30 by expression of a C-terminally haemagglutinin (HA)-tagged version of the protein. We verified that the tagged protein had the correct apparent molecular weight (Fig. 2B), and that TbCAP30-HA localized in the region between the nucleus and the kinetoplast, with signal distribution overlapped with TbEpsinR (Fig. 2C). This supports the possibility that TcCAP30 has the potential to interact with clathrin/EpsinR.

## Discussion

The surface of infectious organisms forms the interface between the pathogen and host and represents the primary target of immune attack. The trypanosome surface composition^36^,^37^ is highly specialised, and the flagellar pocket constitutes a specific region that facilitates efficient internalization of host macromolecules and restricts access of host immune factors to the exposed, endocytic receptors of the parasite^13^,^38^. This paradigm is probably common to all pathogenic trypanosomes, but variation in surface molecules indicates fundamental adaptation to the specific demands of the parasite/host interaction. *In silico* analysis suggests that several major proteins of the endocytic pathway characterised in animals and fungi are absent^16^.

It remains unknown how much diversity is present between the trypanosomatids, but considering the remarkable differences in lifestyles and surface proteins, adaptations are predicted. For example, *T. cruzi* possesses AP-1 to 4, distinct from *Leishmania* which lacks AP-4 and *T. brucei* lacking AP-2. *T. cruzi* also possesses Rab14, which functions in Golgi to endosome transport^39^ and Rab32, which has many roles including phagocytosis^40^; these are additional to the Rab set shared with *T. brucei*^41^. Both Rab14 and Rab32 are present in the last common eukaryotic ancestor, suggesting that *T. brucei* lost these genes, indicating a likely more sophisticated endomembrane system in *T. cruzi*, and providing evidence for significant divergence. Similar variance has been reported in the Apicomplexa^42^.

We exploited two conserved proteins within the clathrin-mediated transport system of *T. cruzi*: the light chain of clathrin (TcCLC) and EpsinR (TcEpsin). We identified cohorts of candidate proteins for both TcCLC and TcEpsinR. The clathrin heavy chain (TcCHC) is the most abundant protein^43^ and other candidate interacting partners appear to be sub-stoichiometric, similar to CCV isolations from metazoa and trypanosomes, reflecting promiscuity of clathrin interactions^19^,^44^,^45^. A range of additional proteins with clear roles in transport also identified.

Surprisingly AP-2 was not present in any of our isolations. While the genes encoding the four subunits of this adaptor complex are absent from the genome of *T. brucei*^34^, they are present in *T. cruzi*^20^,^41^. We predicted AP-2 to be identified, since this complex facilitates clathrin-mediated endocytosis and the pathway is active in *T. cruzi* epimastigotes^20^,^21^. Some unicellular organisms, including yeast, can survive without AP-2^46^,^47^ while very rapid neuronal endocytosis is also AP-2 independent^48^. Specific cargo adaptors support clathrin-mediated endocytosis in the absence of AP-2^49^, and therefore, the AP-2 complex is not mandatory. For *T. brucei* alternate adaptors, such as TbEpsinR and TbCALM, must support clathrin-mediated endocytosis^13^. Since we failed to recover AP-2, but did identify AP-1 and AP-4, this suggests that the result is likely real and unlikely simply failure to maintain clathrin-AP complexes. Therefore the dominant form of endocytosis in *T. cruzi* may be AP-2 independent, suggesting an unexpected mechanistic similarity to African trypanosomes. This is a surprising finding, potentially unifying AP-2 endocytic mechanisms across a broader range of taxa.

In contrast to AP-2, we recovered all AP-1 subunits with both affinity handles. This complex is mainly associated with transport at the *trans-*Golgi network and late endosomes in mammalian cells^49^,^50^ and *T. brucei*^51^,^52^. It is possible that AP-1 has related functions in *T. cruzi,* such as targeting lysosomal enzymes like cruzipain and chagasin^53^ to reservosomes. It is of interest that cruzipain (TcCLB. 507537.20) was also found and that may represent cargo *en route* to the lysosome^54^. The precise function of AP-4 is not well defined^55^, but significantly the ε-subunit of AP-4 complex was also identified in a *T. cruzi* contractile vacuole proteome along with clathrin and AP180^25^, also found here. Significantly, we also recovered Tepsin, a central component of AP-4-containing vesicles^33^. Tepsin and AP-4 have coevolved and organisms lacking AP-4 also lack Tepsin^55^. These data robustly confirm these earlier observations for AP-4. Significantly, we also identified orthologs of TbCAP80 and TbCAP141, recently shown to be involved in endocytosis in African trypanosomes (Manna *et al.*, 2016 under revision), suggesting that these proteins are part of a conserved trypanosome-specific endocytic mechanism.

In conclusion, we report an interactome for clathrin for *T. cruzi*. The cohort contains many highly conserved members, but also several trypanosome-specific factors. Taken together with recent evidence from African trypanosomes, these data indicate the presence of divergent mechanisms for clathrin function in these pathogenic protozoa.

## Materials and Methods

### Parasites

Cultured epimastigote forms of *Trypanosoma cruzi*, clone Dm28c^56^, were grown at 28 °C with weekly passages in liver infusion tryptose (LIT) medium^57^ supplemented with 10% fetal bovine serum (FBS). *T. brucei* bloodstream forms (BSF) strain 427 were maintained in HMI-9 medium supplemented with 10% fetal bovine serum. Cells were subcultured when cell density reached a maximum of 2 × 10^6^ cells.

### Cloning and expression of *Trypanosoma cruzi* TcCLC and TcEpsinR

To generate transgenic epimastigotes stably expressing TcCLC (Clathrin Light Chain, TcCLB.506211.240) with a protein A and C amino-terminal fusion (TcCLC/AC), TcCLC cDNA was cloned into the pTcGWPTP expression vector. The pTcGWPTP vector encodes proteins A and C and is a modification of the previously described pTcGWGFP vector^58^. The TcCLC gene was used to design forward (5′-ATGGACCCTTTTGAAGGAAGC-3′) and reverse (5′-TTATTGAGCGGTTTCGCCCT-3′) primers flanked by sequences compatible with the Gateway (Invitrogen, USA) cloning platform to enable subsequent subcloning into the target vector. The resulting pTcGWPTP plasmid encoding the TcCLC gene fused to proteins A and C was used to transfect parasites.

To generate transfected *T. cruzi* epimastigotes expressing TcEpsinR (epsin-related) with a GFP amino-terminal fusion (TcEpsinR/GFP), TcEpsinR cDNA (TcCLB.506925.70) was cloned into the pTcGWGFP expression vector with resistance to neomicin^58^. This vector was kindly provided by Dr. Michel Batista, Instituto Carlos Chagas/Fiocruz-Paraná, Brazil). The TcEpsinR gene was used to design forward (5′-TCATGAGTATTCCAACCTCCATTCA-3′) and reverse (5′-CCCTCAGACTGTCGGCGCT-3′) primers flanked by sequences compatible with the Gateway cloning platform to enable subsequent subcloning into the target vector (Invitrogen, USA). The resulting pTcGWGFP plasmid encoding the TcEpsinR gene fused to GFP protein was used to transfect parasites.

### *T. cruzi* transfection

*T. cruzi* epimastigote cultures were grown at 28 °C in LIT medium supplemented with 10% FBS to a density of approximately 3 × 10^7^ cells/ml. Parasites were then harvested by centrifugation at 3,000 *g* for 5 min at room temperature, washed once in phosphate-buffered saline (PBS, pH 7.2) and resuspended in 0.4 ml of electroporation buffer (140 mM NaCl, 25 mM HEPES,0.74 mM Na_2_HPO_4_, pH 7.5) at a density of 1 × 10^8^ cells/ml. Cells were then transferred to a cuvette (0.2 cm gap width) and 10-15 μg DNA was added. The mixture was placed on ice for 10 min and then subjected to two pulses of 450 V/500 μF using the Gene Pulser II (Bio-Rad, Hercules, CA, USA). Following electroporation, cells were cultured in 10 ml LIT medium containing 10% FBS and incubated for 24 h at 28 °C. The antibiotic G418 (500 μg/ml) was then added to the culture medium and stable, resistant cells were obtained approximately 20 days after transfection. Stably transfected cells were maintained in cultures containing 250 μg/ml G418.

### One-step PCR-mediated transfection of *T. brucei* BSF cells for *in-situ* tagging

To generate transfected *T. brucei* BSF cells expressing TbCAP30 with a 3xHA carboxy-terminal fusion (TbCAP30/HA), TbCAP30 cDNA (Tb927.8.7230) was cloned into the pMOTag2H (kindly provided by George Cross, Addgene plasmid #26296) with a puromycin selectable marker and a 3xHA-tag^59^. The TbCAP30 gene sequence was used to design forward (5′-GTTGACGTTGACCGTGTTTACGTACCAGGGACGGTGGAGGCCGCTAAGGCGCTCGGCACTTCTGAGAAGCAGGGGTACAATGCGGTTGTTGGTACCGGGCCCCCCCTCGAG-3′) and reverse (5′-TGCCCATTTCAACCGCTTTCACTGCTTGCCCTTTCCCTTTTCCCCTCTTTCTTTATATATATATATATATATCCCCAACCTTCCTCGAAGTGGCGGCCGCTCTAGAACTAGTGGAT-3′) primers. By using one-step PCR the 3′ UTR of the target gene was replaced by a heterologous intergenic region. This replacement directs the correct splicing of the downstream antibiotic resistance marker.

*T. brucei* transfection: At a cell density of 1.0–1.5 × 10^6^ cells/ml, 3.5 × 10^7^ BSF were harvested by centrifugation for 10 minutes, 800 *g* at 4°C. The supernatant was removed and the cells resuspended in 100 μl of Amaxa buffer (Lonza; Basel, Switzerland), mixed with 30 μg of ethanol precipitated linear PCR product and transferred into a sterile cuvette. Electroporation was performed using an Amaxa Nucleofector II (Lonza) as described. Cells were immediately transferred into pre-warmed HMI-9 and cultured at 37 °C to recover. After 6 hours, the antibiotic puromycin (2 μg/ml) was added and the cells were transferred to 24 well plates to enable the isolation of clonal-antibiotic resistant populations after 7–14 days, and were further expanded in continuous presence of antibiotic.

### Immunofluorescence in *T. cruzi*

For colocalisation of endogenous TcCHC (clathrin heavy chain) with exogenously expressed TcEpsin/GFP in transfected *T. cruzi* epimastigotes, 3-day-old cells were washed twice with PBS, fixed for 30 min with 4% paraformaldehyde and adhered to poly-L-lysine coated slides and incubated for 1 h at 37 °C with anti-GFP antibody (1:100) and TcCHC monoclonal antibody^21^. After three washes in PBS, the samples were incubated under the same conditions with a secondary Alexa Fluor 488-conjugated goat anti-rabbit antibody (1:600) and an Alexa Fluor 594-conjugated anti-mouse antibody (1:600). A negative control was performed by incubating anti-GFP antibody with wild-type epimastigotes (data not shown). Nuclear and kinetoplast DNA were stained with Hoechst 33342. After extensive washes, the slides were prepared with mounting medium containing N-propyl-gallate as an anti-fade agent. The samples were examined using a Leica SP5 confocal laser-scanning microscope (Leica Microsystems, Mannheim, Germany) at the Microscopy Facility of the Carlos Chagas Institute, Fiocruz-PR. Acquired images were processed for presentation using Adobe Photoshop CS5 (Adobe Systems Incorporated, USA).

### Immunofluorescence in *T. brucei*

Mid-log phase cells were harvested and washed with Voorheis’ PBS (PBS supplemented with 10 mM glucose and 46 mM sucrose, pH 7.6: vPBS). The cells were subsequently fixed in 4% paraformaldehyde (w/v) and adhered to poly-L-lysine coated slides. For permeabilization and staining of internal structures, cells were incubated with 0.1% Triton X-100 (v/v) in vPBS, washed with vPBS and blocked with 20% fetal bovine serum in PBS. For co-staining, the fixed cells were incubated with a polyclonal rabbit antiserum anti-TbEpsinR conjugated to AlexaFluor 488 and a rat antibody against HA conjugated to AlexaFluor 594. The slides were dried and mounted with a drop of Vectashield supplemented with 4,6-diamidino-2-phenylindole (DAPI) (Vector Laboratories, USA) to stain DNA. Images were acquired on a Nikon Eclipse E600 epifluorescence microscope with a Hammamatsu ORCA CCD camera and images captured using Metamorph software. Final processing for presentation was done using Adobe Photoshop CS5 (Adobe Systems Inc.).

### Cryomilling

To identify the proteins associated with clathrin in coated vesicles, *T. cruzi* epimastigotes expressing TcCLC/AC, *T. cruzi* epimastigotes expressing TcEpsinR/GFP and *T. cruzi* wild-type epimastigotes were submitted to cryomilling with subsequent immunoprecipitation of associated complexes^32^. This method requires substantial quantities of starting material, but allows retention of protein-protein interactions not otherwise preserved. Briefly, a total of 5 × 10^10^ cells were harvested by centrifugation at 3000 *g* for 10 s and the cells snap frozen in liquid nitrogen and milled using a ball mill in liquid nitrogen (Retsch Planetary Ball Mill PM100, Haan, Germany) to produce a cryogrindate, under essentially native conditions.

### Immunoprecipitation and identification of TcCLC associated proteins by mass spectrometry (MS)

A total of 350 μg of cell powder was resuspended in 1 ml of CHC buffer (20 mM Hepes 7.4, 250 mM citrate, 0.1% CHAPS, 1 mM MgCl_2_ 10 μM CaCl_2_, plus protease inhibitor cocktail) and complexes were subsequently bound to 350 μl of Dynabeads M280 coupled to sheep anti rabbit-IgG (Life Technologies, USA). A grindate prepared from wild type cells was used as a negative control. After incubation the beads were washed in the same buffer and eluted in 50 μl of elution buffer (20 mM Tris pH 8, 2% SDS) for 30 min at 72 °C. From the supernatant 5 μl were used to SDS-PAGE, stained with Coomassie and 5 μl Western blotted. To the remaining 40 μl, 427 μl of ethanol was added and incubated for 16 h at −20 °C and then the sample was centrifuged at 20,000 *g* for 30 minutes at 4 °C. The resulting pellet was analysed by LCMS^2^.

### Reverse co-immunoprecipitation (TcEpsinR/GFP)

Immunoprecipitation using a llama polyclonal anti-GFP antibody was performed using *T. cruzi* epimastigotes expressing TcEpsinR/GFP. For TcEpsinR immunoisolation, 350 μg of cell grindate was ressuspended in CHC Buffer and complexes were subsequently bound to 35 μl of Dynabeads M270 epoxy coupled to llama anti-GFP. After incubation the beads were washed in the same buffer and then processed as described above.

### Mass spectrometry

Liquid chromatography tandem mass spectrometry (LC-MS/MS) was performed by the Proteomic Facility at the University of Dundee. To separate proteins for mass spectrometry analysis, the samples were run 2 cm on a 10% SDS gel (NuPAGE^®^ Bis-Tris 10% gels, Novex by Life Technologies) in a 1× MOPS SDS running buffer, fixed and stained with Coomassie. The selected 2 cm gel piece was excised and in-gel tryptic digestion (Trypsin, Modified Sequencing Grade, Roche) was carried out for 16 h at 37 °C. Peptides were extracted with 0.1% trifluoroacetic acid in 50% acetonitrile and dried in a SpeedVac. Peptides were then resuspended in 1% formic acid, centrifuged (13,000 rpm, 1 min) and transferred to an HPLC (high performance liquid chromatography) vial. Usually, 5 μl of this suspension was analysed. Samples were analysed using an Ultimate 3000 RSLC nano system coupled on-line to a LTQ OrbiTrap Velos Pro equipped with an Easy-Spray source (Thermo Scientific). Peptides were initially trapped and desalted using an Acclaim^®^ PepMap100 C18 Nano-trap column (100 μM × 2 cm) with 0.1% formic acid (buffer A). After 3 min, a wash gradient was formed to separate the peptides using a 180 min gradient on an Easy-Spray PepMap RSLC C18 column (75 μM × 50 cm). Samples were transferred to the mass spectrometer via an Easy-Source with the temperature set at 50 °C and a source voltage of 1.9 kV. The mass spectrometer was operated in standard data dependent acquisition mode. Survey full scan MS spectra were acquired with a resolution of 60,000 at m/z 335-1800. The AGC was set to 1 × 10^6^ and an ion trap Msn target value of 5000 was used. The top 15 most intense ions were targeted for CID fragmentation (2 Da isolation window), with normalized collision energy of 35% in the linear ion trap. The dynamic exclusion time window was set to 45 sec, with an isowidth of 2 Da. Once part of the mass range has been excluded for the set time it is released again^60^. Lock mass of 445.120024 was enabled for all experiments.

The mass spectra was analyzed using the Mascot search engine tool (Version 2.3.2) (http://www.matrixscience.com/) against the database of protein sequences from *T. cruzi* UniProt (54,500 sequences) of five different strains of *T. cruzi* (CL Brener Esmeraldo-like, CL Brener non Esmeraldo-like, Sylvio, Dm28c and Marinkellei). This strategy was used to increase the coverage of identified peptides. The abundance of proteins was deduced from the total number of MS /MS spectra generated from the same related peptides^61^. The approximate relative quantification of these proteins in complex was estimated in label-free mode and through the exponentially modified protein abundance index (emPAI)^62^.

### Relative quantitative real time (qRT)-PCR

Total RNA was extracted using the RNeasy mini kit (Qiagen) according to the manufacturer’s instructions along with DNase treatment and quantified using a ND-1000 spectrophotometer and Nanodrop software (Nanodrop Technologies). For cDNA synthesis, 2 μg RNA was diluted to 10 μl with diethylpyrocarbonate (DEPC)-treated water and denatured at 70 °C, 5 min. 15 μl of a reaction mix was added (2.5 μl dNTPs (25 mM stock), 5 μl 5× reverse transcription buffer (Invitrogen), 2 μl 100 mM DTT, 0.5 μl RNAseOUT (recombinant ribonuclease inhibitor, 5000 U/μl, Invitrogen), 2 μl oligo dT, (T_30_VN, 10 μM stock) 0.5 μl Superscript II Reverse Transcriptase (200 U/μl Invitrogen), and 2.5 μl DEPC-treated water and incubated at 37 °C for 1 hr, heat-inactivated at 90 °C, 5 min and finally diluted to 200 μl with DEPC-treated water. For qRT-PCR, 5 μl of cDNA was used in a 25 μl reaction including IQ SYBR Green Supermix (BioRad) with 0.4 μM gene-specific forward and reverse primers. qRT-PCR reactions were performed in white thin wall polypropylene multiplate 48-well unskirted PCR plates (BioRad) sealed with microseal ‘B’ adhesive (BioRad). Reactions were performed in a BioRad MiniOpticon real time PCR detection system and included an initial denaturation at 95 °C for 3 min, 40 cycles of 95 °C 30 seconds, 58°C 30 sec, 72 °C 30 sec (with a signal read at the end of each cycle). In each amplification step, a non-template control was subjected to the reaction to ensure that there was no contamination.

### Comparative genomics

The predicted sequences of TcCLC and TcEpsinR-associated proteins were obtained using the Tritryp database (http://tritrypdb.org/tritrypdb/). AP180, CAP30, CAP37, CAP80, CAP141, GLP-1 and Tepsin were selected as query sequences in DELTA-BLAST interrogations. A broad range of 18 eukaryotic genomes was inspected: *Arabidopsis thaliana*, *Batrachochytrium dendrobatidis*, *Bigelowiella natans*, *Bodo saltans*, *Chlamydomonas reinhardtii*, *Cyanidioschyzon merolae*, *Dictyostelium discoideum*, *Drosophila melanogaster*, *Entamoeba histolytica*, *Homo sapiens*, *Leishmania major*, *Naegleria gruberi*, *Phytomonas serpens*, Saccharomyces cerevisiae, *Tetrahymena thermophilia*, *Toxoplasma gondii*, *T. brucei* and *T. cruzi*, spanning this domain diversity at NCBI (http://www.ncbi.nlm.nih.gov/BLAST/). The best returned candidate orthologs were recipriocal BLASTed against the protein database of *T. cruzi*. Proteins that retrieved the original query sequence were further considered, taking into account the presence of conserved domains using pfam and NCBI CDDB with default parameters. Lastly, both query and subject sequences were aligned with Clustal in order to access identity and to inspect for homology across the entire predicted sequence. Data are displayed as a Coulson plot using Coulson Plot Generator v1.4.7^63^ .

## Additional Information

**How to cite this article**: Kalb, L. C. *et al.* Conservation and divergence within the clathrin interactome of *Trypanosoma cruzi.* *Sci. Rep.* **6**, 31212; doi: 10.1038/srep31212 (2016).

## Supplementary Material

Supplementary Dataset 1

Supplementary Dataset 2

## Figures and Tables

**Table 1:** TriTrypDB accessions and annotations for TcCLC-associated proteins identified from mass spectrometry.

Accession	Annotation	Rep 1	Con 1	Rep 2	Con 2
Clathrin					
**TcCLB.506167.50**	**Clathrin HC**	**268,81**	**0,86**	**54,01**	**0,19**
**TcCLB.506211.240**	**Clathrin LC**	**15,81**	**0**	**2,17**	**0**
**TcCLB.510045.30**	**Auxilin**	**2,78**	**0**	**0,5**	**0**
**Adaptor complex 1**					
**TcCLB.508257.260**	**AP-1γ**	**1,78**	**0**	**0,68**	**0**
**TcCLB.506247.200**	**AP-1β**	**1,65**	**0,2**	**0,15**	**0**
**TcCLB.510533.40**	**AP-1μ**	**0,96**	**0**	**0,53**	**0**
**TcCLB.509623.19**	**AP-1σ**	**0,36**	**0**	**0**	**0**
**Adaptor complex 4**					
**TcCLB.511751.200**	**AP-4ε**	**0,98**	**0**	**0,12**	**0**
TcCLB.506525.104	AP-4σ	0,77	0	0	0
TcCLB.504137.60	AP-4β	0,74	0	0,07	0
**TcCLB.509911.70**	**AP-4μ**	**0,7**	**0**	**0,11**	**0**
**Other adaptors**					
**TcCLB.506925.70**	**EpsinR**	**12,11**	**0,9**	**3,03**	**0**
**TcCLB.504105.120**	**Tepsin**	**5,7**	**0**	**1,2**	**0**
**TcCLB.503449.30**	**AP180/CALM**	**1,15**	**0**	**0,24**	**0**
**SNAREs**					
**TcCLB.508465.120**	**Syntaxin 16**	**1,15**	**0**	**0**	**0**
**TcCLB.508955.10**	**Qc**	**0,41**	**0**	**0**	**0**
**TcCLB.506855.140**	**Vamp7c**	**0**	**0**	**0,25**	**0**
**TcCLB.507795.50**	**Syntaxin 7**	**0**	**0**	**0,22**	**0**
**Rabs**					
**TcCLB.509805.60**	**Rab5**	**0,64**	**0**	**0**	**0**
TcCLB.508461.270	Rab7	0,36	0	0,23	0,11
TcCLB.511621.120	Rab14	0,87	0,46	0,29	0
TcCLB.511711.80	Rab2	0,3	0		
**Scission proteins**					
TcCLB.508153.20	Dynamin	1,41	0	0	0
**Cargo proteins**					
**TcCLB.511391.180**	**GLP-1**	**3,92**	**1,12**	**1,12**	**0,05**
TcCLB.507537.20	Cruzipain	0,53	0	0,13	0
**Trypanosome-specific clathrin-associated proteins**					
**TcCLB.503595.10**	**CAP80**	**0,92**	**0**	**0,18**	**0**
**TcCLB.507221.70**	**CAP141**	**1,81**	**0**	**0,14**	**0**
TcCLB.510057.30	CAP37	0	0	1,25	0
**Others**					
**TcCLB.509319.40**	**DUF846**	**0**	**0**	**0,24**	**0**
**TcCLB.503791.49**	**Vps45**	**1,25**	**0**	**0,1**	**0**

The emPai scores for three independent replicate (Rep) isolations are shown in columns C to F together with concurrent control isolations using cryolysates from untagged cells under the same buffer conditions. Isolation buffer used was 20 mM Hepes 7.4, 250 mM citrate, 0.1% CHAPS, 1 mM MgCl_2_ 10 μM CaCl_2_, plus protease inhibitor cocktail. Accessions in bold are in common with the TbEpsinR isolation (Table 2). Only proteins identified with a five-fold greater emPai against the control and greater than 0.1 are shown.

**Table 2:** TriTrypDB accessions and annotations for TcEpsinR-associated proteins identified from mass spectrometry.

Accession	Annotation	Rep 1	Con1	Rep 2	Con 2	Rep 3	Con 3
Clathrin							
**TcCLB.506167.50**	**Clathrin HC**	**82,17**	**0**	**26,32**	**0,59**	**21,94**	**0,49**
**TcCLB.506211.240**	**Clathrin LC**	**1,79**	**0**	**2,61**	**0**	**3,66**	**0,14**
**TcCLB.510045.30**	**Auxilin**	**0,27**	**0**	**0**	**0**	**0,17**	**0**
**Adaptor complex 1**							
**TcCLB.508257.260**	**AP-1γ**	**1,71**	**0**	**1,45**	**0,32**	**1,06**	**0,23**
**TcCLB.506247.200**	**AP-1β**	**0,88**	**0**	**1,36**	**0**	**0,93**	**0**
**TcCLB.510533.40**	**AP-1μ**	**0,85**	**0**	**1,22**	**0**	**1,09**	**0,2**
**TcCLB.509623.19**	**AP-1σ**	**0,36**	**0**	**0,59**	**0**	**0,17**	**0**
**Adaptor complex 4**							
**TcCLB.511751.200**	**AP-4ε**	**0,11**	**0**	**0,12**	**0**	**0**	**0**
**TcCLB.509911.70**	**AP-4μ**	**0,05**	**0**	**0,24**	**0**	**0,11**	**0**
**Other adaptors**							
**TcCLB.506925.70**	**EpsinR**	**12,83**	**0**	**17,08**	**0**	**13,59**	**0**
**TcCLB.504105.120**	**Tepsin**	**0,39**	**0**	**1,2**	**0**	**1,2**	**0**
**TcCLB.503449.30**	**AP180/Calm**	**0,18**	**0**	**0,12**	**0**	**0**	**0**
**SNAREs**							
TcCLB.511627.60	VAMP7a	1,26	0	0,59	0	0,26	0
**TcCLB.506855.140**	**Vamp7c**	**0,74**	**0**	**0,95**	**0**	**0,95**	**0**
TcCLB.507811.60	Vamp7b	0,45	0	0,85	0	0,85	0
**TcCLB.507795.50**	**Syntaxin 7**	**0,22**	**0**	**0,11**	**0**	**0,22**	**0**
**TcCLB.508955.10**	**Qc**	**0,22**	**0**	**0,58**	**0**	**0,41**	**0**
TcCLB.506401.130	Qa	0,16	0	0,57	0	0	0
**TcCLB.508465.120**	**Syntaxin 16**	**0,09**	**0**	**0,53**	**0,09**	**0,29**	**0**
**Rabs**							
**TcCLB.509805.60**	**Rab5**	**0**	**0**	**0,64**	**0**	**0,85**	**0**
TcCLB.510911.30	Rab4	0,49	0	0	0	0,49	0
**Cargo proteins**							
**TcCLB.511391.180**	**GLP-1**	**0,19**	**0**	**2,77**	**0,42**	**3,5**	**0,42**
**Recycling system**							
TcCLB.506925.100	SCAMP domain	0,15	0	0	0	0,15	0
**Trypanosome specific clathrin-associated proteins**							
**TcCLB.503595.10**	**CAP80**	**0,18**	**0**	**0,28**	**0**	**0,13**	**0**
**TcCLB.507221.70**	**CAP141**	**0,18**	**0**	**0,26**	**0**	**0,22**	**0**
TcCLB.507895.170	CAP30	0,22	0	0,22	0	0,22	0
**Others**							
**TcCLB.509319.40**	**DUF846**	**0,24**	**0**	**0,53**	**0**	**0,53**	**0**
**TcCLB.503791.49**	**Vps45**	**0,21**	**0**	**0,4**	**0**	**0,47**	**0**

The emPai scores for three independent replicate (Rep) isolations are shown in columns C to H together with concurrent control isolations using cryolysates from untagged cells under the same buffer conditions. Isolation buffer used was 20 mM Hepes 7.4, 250 mM citrate, 0.1% CHAPS, 1 mM MgCl_2_ 10 μM CaCl_2_, plus protease inhibitor cocktail. Accessions in bold are in common with the clathrin light chain isolation (Table 1). Only proteins identified with a five-fold greater emPai against the control and greater than 0.1 are shown.
